# Changes in aberrations and biomechanics after femtosecond laser-assisted laser in situ keratomileusis (FS-LASIK) in eyes with high astigmatism: a retrospective case control study

**DOI:** 10.1186/s12886-023-02809-4

**Published:** 2023-02-13

**Authors:** Na Li, Tong Chen, Ge Tian, Yue Lin, Yuan Meng, Hua Gao, Mingna Liu

**Affiliations:** 1 Eye Institute of Shandong First Medical University, Eye Hospital of Shandong First Medical University (Shandong Eye Hospital), 372 Jingsi Road, 250021 Jinan, China; 2 State Key Laboratory Cultivation Base, Shandong Provincial Key Laboratory of Ophthalmology, 372 Jingsi Road, 250021 Jinan, China; 3 School of Ophthalmology, Shandong First Medical University, 372 Jingsi Road, 250021 Jinan, China

**Keywords:** Aberrations, Biomechanics, Femtosecond laser-assisted laser in situ keratomileuses, High astigmatism

## Abstract

**Background:**

To compare the 6-month changes in aberration and biomechanics after femtosecond laser-assisted laser in situ keratomileusis (FS-LASIK) for high astigmatism.

**Methods:**

In this retrospective case control study, 47 eyes with high astigmatism (≥ 2.5 D, HA group) and 47 eyes with low astigmatism (≤ 1.0 D, LA group) underwent FS-LASIK. Preoperative and follow-up examinations included visual outcomes, higher order aberrations (HOAs) and biomechanics. Biomechanical parameters include a deformation amplitude ratio of 2 mm (DA ratio 2 mm), integrated inverse radius (IIR), stiffness parameter at first applanation (SP-A1), and ambrosio relational thickness through the horizontal meridian (ARTh).

**Results:**

Six months postoperatively, there was no significant difference in the efficacy and safety index (both *P* > 0.05) between the two groups, but the cylinder was higher in the HA group. The HOAs increased significantly after surgery in both groups (all *P* < 0.05). Six months postoperatively, the changes in spherical aberration and HOAs were larger in the HA group (both *P* < 0.005), but there was no significant difference between the changes in coma (*P* > 0.05). Significant decreases in SP-A1 and ARTh and significant increases in the IIR and DA ratio of 2 mm (all *P* < 0.05) were observed after surgery in both groups. The changes in the DA ratio 2 mm, IIR, SP-A1, and ARTh were not significantly different between the groups.

**Conclusion:**

FS-LASIK had relative comparable efficacy and safety in correcting high and low myopic astigmatism, with higher astigmatic under-correction in eyes with high astigmatism. High astigmatism in eyes after FS-LASIK could introduce larger corneal aberrations, but the impact on corneal stiffness was the same as that in eyes with low astigmatism.

## Background

Femtosecond laser-assisted laser in situ keratomileusis (FS-LASIK) was first reported by Ratkay-Traub et al. in 2003 [1]. This procedure has many advantages over a mechanical microkeratome and fewer side effects, such as free caps, irregular flaps, and buttonholes, etc. [2–4] Many studies have shown the good safety, efficacy, and predictability in FS-LASIK, including in eyes with high myopia or high astigmatism [5–7].

In principle, corneal refractive surgery aims to correct refractive errors by ablating a certain amount of stroma in the central optical zone of the cornea. Central optical ablation of the corneal stroma is performed exclusively on myopic eyes, while a hyperopic/astigmatic treatment involves mid-peripheral ablation, and a higher degree of refractive error requires deeper ablation. Corneal ablation inevitably causes thinning and flattening of the cornea; the former leads to a significant reduction in corneal biomechanics [8, 9], while the latter introduces higher-order aberrations (HOAs) of the cornea [10, 11].

Several studies have reported that HOAs and biomechanical changes induced by surgical operations are more considerable in the high myopia group than in the low to moderate myopia group for the removal of more tissue [12–14]. However, changes in HOAs and biomechanics after FS-LASIK in eyes with high astigmatism have rarely been reported. This retrospective study aimed to compare changes in aberration and biomechanics in patients with high myopic astigmatism (≥ 2.5 D) and low myopic astigmatism (≤ 1.0 D) after FS-LASIK in 6 months follow-up study.

## Methods

### Patients

This study included 94 patients with myopic astigmatism of 2.5 D (2.5 D was selected according to Varma R.’s [15] report, which defined high astigmatism as over 2.25 D) or more and 1.0 D or less who underwent FS-LASIK between September 2019 and September 2021 at Shandong Eye Hospital. This retrospective study was approved by the Institutional Review Board of the Eye Hospital of Shandong First Medical University. This study adhered to the tenets of the Declaration of Helsinki. All patients provided written informed consent for their medical information to be included in this study.

The inclusion criteria were age ≥ 18 years, stable myopia and astigmatism, or a minor change in 0.50 D at least 12 months. Patients with suspected keratoconus based on corneal topography, severe dry eyes, ocular inflammation, infection, systemic diseases, immune system diseases, depression, and pregnancy were excluded from the study.

### Surgical techniques

All the procedures were performed by a single surgeon (H. Gao). In the FS-LASIK procedure, a lamellar flap was created using a femtosecond laser system (Carl Zeiss Meditec AG, Jena, Germany). The flaps had diameters of 8.1 mm and thicknesses of 100 μm with superior hinges and 90-degree side-cut angles. The laser separation distance of the flap and flap side was 4.5 μm and 2.0 μm. After flap creation, a spatula was used to pass across the flap from the hinge and sweep inferiorly to lift the flap for excimer laser ablation. Stromal tissue ablation was performed with the Allegretto Wave excimer laser system WaveLight Laser Technologie AG (Alcon Laboratories, Fort Worth, TX, USA), standard aspheric (Wavefront Optimized), with a flying spot laser of 0.95 mm in diameter, a repetition rate of 500-Hz, and an active video-based 1050-Hz eye tracker, and the optical zone of ablation was between 6.00 and 6.50 mm, depending on the central corneal thickness and the refractive error to be corrected. The flaps were repositioned after excimer laser treatment.

### Preoperative and postoperative assessment

Before surgery, all patients underwent a detailed ophthalmological examination that included evaluation of the uncorrected (UDVA) and corrected distance visual acuity (CDVA) of the logarithm of the minimum angle of resolution (logMAR), intraocular pressure, manifest refraction, slit-lamp examination keratometry, corneal tomography (Pentacam HR; Oculus Optikgeräte GmbH, Wetzlar, Germany), and dynamic Scheimpflug analysis (Corvis ST II, Oculus Optikgeräte GmbH, Wetzlar, Germany). All examinations were repeated 1, 3, and 6 months postoperatively. The following Pentacam HR parameters were registered for the anterior cornea in a central zone of 6 mm: root mean square (RMS) of coma, RMS of spherical aberration, and RMS of higher-order aberrations. The changes (Δ) in HOAs parameters were calculated as postoperative values minus preoperative values, and the changes in biomechanical parameters were calculated as preoperative values minus postoperative values. All measurements were performed by the same investigator to eliminate any possible inter-observer variability. Each measurement was performed in triplicate to ensure good repeatability and the average value was used in the analysis.

### Statistical analysis

Data are presented as mean ± standard deviation (SD). An independent t-test was used to compare continuous variables between the two groups. An analysis of covariance (ANCOVA) was used to adjust for different pre-existing pre-spheres of the baseline. Categorical variables were assessed using Pearson’s chi-square test. Repeated-measures analysis of variance was applied to compare the variables at each follow-up. Fisher’s least significant difference (LSD) test was used for multiple comparisons within each group. The safety index was equal to the ratio of postoperative CDVA (logMAR) to preoperative CDVA (logMAR), and the efficacy index was equal to the ratio of postoperative UDVA (logMAR) to preoperative CDVA (logMAR). Statistical analysis was performed using the SPSS software (version 22.0). A *P*-value of less significance was set at p < 0.05.

## Results

A total of 47 subjects (21 females, 26 males; 19 right eyes, 28 left eyes) with astigmatism higher than 2.5 D were included in this study (HA group, mean cylinder: -3.24 ± 0.68 D, range: -5.25 ~ − 2.50 D) and 47 subjects (24 females, 23 males; 23 right eyes, 24 left eyes) with astigmatism lower than 1.0D (LA group, mean cylinder: -0.36 ± 0.36 D, range: −1.0 ~ 0 D) served as controls. Table 1 shows patient characteristics by groups. The mean preoperative spherical error was higher in the LA group (− 5.96 ± 1.65 for HA and − 6.81 ± 1.67 for LA, *P* = 0.016) and showed no significant differences in the preoperative SEQ between the two groups (− 7.58 ± 1.64 for HA and − 6.99 ± 1.72 for LA, *P* = 0.094). Eyes with low astigmatism had a significantly thinner preoperative CCT (*P* = 0.020, Table 1) than eyes with high astigmatism. No intraoperative complications occurred during any surgery.

**Table 1:** Preoperative characteristics (mean ± SD) of eyes in HA group and LA group

Characteristics	HA group	LA group	*P*
No. of eyes (R/L)	47 (19/28)	47 (23/24)	0.412
Sex (F/M)	21/26	24/23	0.541
Age (y)	21.64 ± 4.42	23.40 ± 4.51	0.061
CDVA (logMAR)	0.00 ± 0.03	-0.01 ± 0.03	0.195
bIOP (mmHg)	16.54 ± 2.26	16.90 ± 2.28	0.447
Refractive errors (D)			
Sphere	-5.96 ± 1.65	-6.81 ± 1.67	0.016*
Cylinder	-3.24 ± 0.68	-0.36 ± 0.36	< 0.000*
Spherical equivalent	-7.58 ± 1.64	-6.99 ± 1.72	0.094
Central corneal thickness (µm)	553.49 ± 27.42	539.53 ± 28.98	0.020*

SD, standard deviation; HA, high astigmatism; LA, low astigmatism; R, right eye; L, left eye; M, male; F, female; y, year; CDVA, corrected distance visual acuity; logMAR, logarithm of the minimum; bIOP, biomechanically-corrected intraocular pressure measurements; D, diopters; *P < 0.05

**Fig. 1:**
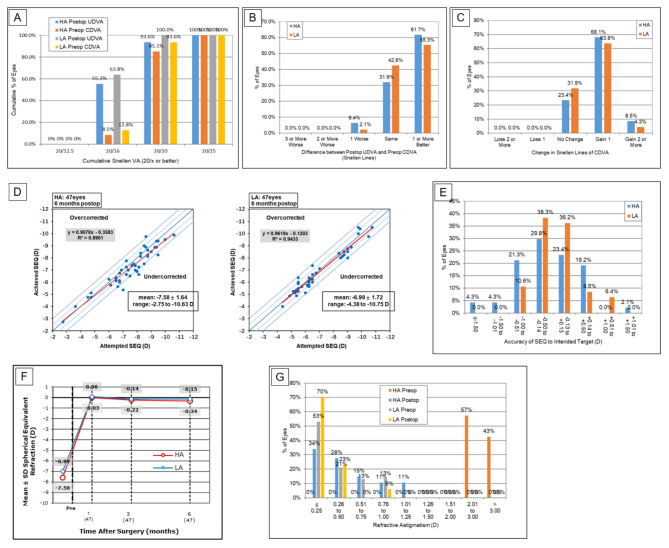
Visual outcomes after FS-LASIK for high and low astigmatism eyes (A) Cumulative 6-month postoperative uncorrected distance visual acuity (UDVA) and preoperative corrected distance visual acuity (CDVA). Changes in Snellen lines of (B) postoperative UDVA and (C) CDVA relative to preoperative CDVA. (D) The attempted versus achieved changes in spherical equivalent refraction (SEQ) and (E) the accuracy of SEQ to the intended target at 6 months after surgery. (F) The SEQ stability and (G) the comparative distribution of preoperative and 6-month postoperative cylinder at 6 months after surgery. D = diopters

Table 2 shows the postoperative characteristics of eyes in the HA and LA groups. Although the eyes with low astigmatism had a significantly thinner ablation depth (*P* = 0.002, Table 2) and larger optical zone (*P* < 0.001, Table 2) than the eyes with high astigmatism, the percent of tissue altered (*P* = 0.195, Table 2) and the mean residual stromal bed thickness (P = 0.459, Table 2) were not statistically different between the groups. At the 6-month follow-up, there were no significant differences in UDVA (*P* = 0.174), CDVA (*P* = 1.000), efficacy index (*P* = 0.782), and safety index (*P* = 0.303). As shown in Figs. 1A, 44 eyes (93.6%) in the HA group and 47 eyes (100%) in the LA group had a UDVA ≥ 20/20 (*P* = 0.174). Figure 1B shows that 93.6% of patients in the HA group and 97.9% of patients in the LA group had postoperative UDVA equal to or better than the preoperative CDVA (*P* = 1.000). None of the eyes in the HA or LA groups lost one or more lines post-CDVA. (Fig. 1C)

**Table 2:** Postoperative characteristics of eyes in both groups at 6 months follow-up

Characteristics	HA group	LA group	*P*
Ablation depth (µm)	106.09 ± 16.51	96.26 ± 12.32	0.002*
Percent tissue altered	0.37 ± 0.03	0.36 ± 0.03	0.195
Residual stromal bed thickness (µm)	340.72 ± 28.29	339.04 ± 27.12	0.459
Optical zone (mm)	6.00 ± 0.00	6.35 ± 0.23	< 0.001*
UDVA (logMAR)	-0.04 ± 0.05	-0.05 ± 0.04	0.174
CDVA (logMAR)	-0.06 ± 0.03	-0.06 ± 0.03	1.000
Safety Index	1.16 ± 0.11	1.14 ± 0.10	0.303
Efficacy Index	1.10 ± 0.15	1.11 ± 0.11	0.782
Refractive errors (D)			
Sphere	-0.07 ± 0.55	-0.01 ± 0.43	0.533
Cylinder	-0.54 ± 0.39	-0.28 ± 0.26	< 0.001*
Spherical equivalent	-0.34 ± 0.54	-0.15 ± 0.41	0.057
Attempted	-7.58 ± 1.64	-6.99 ± 1.72	0.094
Achieved	-7.24 ± 1.58	-6.84 ± 1.70	0.247

HA, high astigmatism; LA, low astigmatism; UDVA, uncorrected distance visual acuity; CDVA, corrected distance visual acuity; logMAR, logarithm of the minimum; D, diopters. *P < 0.05

In the 6-month follow-up period, there was no significant difference in the SEQ (*P* = 0.057) or sphere (*P* = 0.533), but there was a significant difference in the cylinder (*P* < 0.001). Figure 1D shows a scatter plot of attempted versus achieved SEQ refraction in the HA and LA groups. Within ± 0.50 D of emmetropia, 34 eyes (72.3%) in the HA group were comparable with 39 eyes (83.0%) in the LA group (*P* = 0.322). Forty-four eyes (93.6%) in the HA group and 47eyes (100%) in the LA group were comparable within ± 1.00 D (*P* = 0.241; Fig. 1E). There was no statistically significant difference in the SEQ in the HA and LA groups throughout the 1 − 6 months postoperative period (*P* = 0.136; Fig. 1F), which suggested the stability of refraction in both groups. Figure 1G shows the astigmatism amplitude, both postoperatively and preoperatively. In 16 eyes (34.0%) in the HA group and 33 eyes (70.2%) in the LA group, it was less than or equal to 0.25 D cylinder (*P* = 0.001). Correspondingly, 29 eyes (61.7%) and 44 eyes (93.6%) had a postoperative cylinder ≤ 0.50 D (*P* < 0.001), and 42 eyes (89.4%) and 47 eyes (100%) had a postoperative cylinder ≤ 1.00 D (*P* = 0.066).

Spherical aberration, coma, and HOAs increased significantly after surgery in both groups. Six months postoperatively, spherical aberration (0.660 ± 0.224 *VS* 0.456 ± 0.235) and HOAs (1.145 ± 0.382 *VS* 0.872 ± 0.331) were higher in the HA group than those in the LA group (both *P* < 0.001), and the postoperative changes of spherical aberration (0.434 ± 0.204 *VS* 0.254 ± 0.241) and HOAs (0.699 ± 0.329 *VS* 0.494 ± 0.341) in the HA group were also higher than those in the LA group (both *P* < 0.005). However, there were no significant difference between groups was found in the coma values (*P* = 0.707) or the change in coma (*P* = 0.628) 6 months after surgery. Table 3.

**Table 3:** Comparison of HOAs between the groups before and 6 months after FS-LASIK

Parameters	RMS of spherical aberration			RMS of coma			RMS of HOAs		
	HA group	LA group	*P* value	HA group	LA group	*P* value	HA group	LA group	*P* value
Preoperative	0.226 ± 0.096	0.202 ± 0.086	0.219	0.240 ± 0.152	0.177 ± 0.089	0.019*	0.446 ± 0.214	0.378 ± 0.087	0.047*
6 months	0.660 ± 0.224	0.456 ± 0.235	< 0.001*	0.618 ± 0.366	0.590 ± 0.332	0.707	1.145 ± 0.382	0.872 ± 0.331	< 0.001*
*P* value	< 0.001*	< 0.001*	--	< 0.001*	< 0.001*	--	< 0.001*	< 0.001*	--
Changes (Δ)	0.434 ± 0.204	0.254 ± 0.241	< 0.001*	0.378 ± 0.330	0.413 ± 0.356	0.628	0.699 ± 0.329	0.494 ± 0.341	0.004*

HOA, higher-order aberration; RMS, root mean square; HA, high astigmatism; LA, low astigmatism. *P < 0.05

**Fig. 2:**
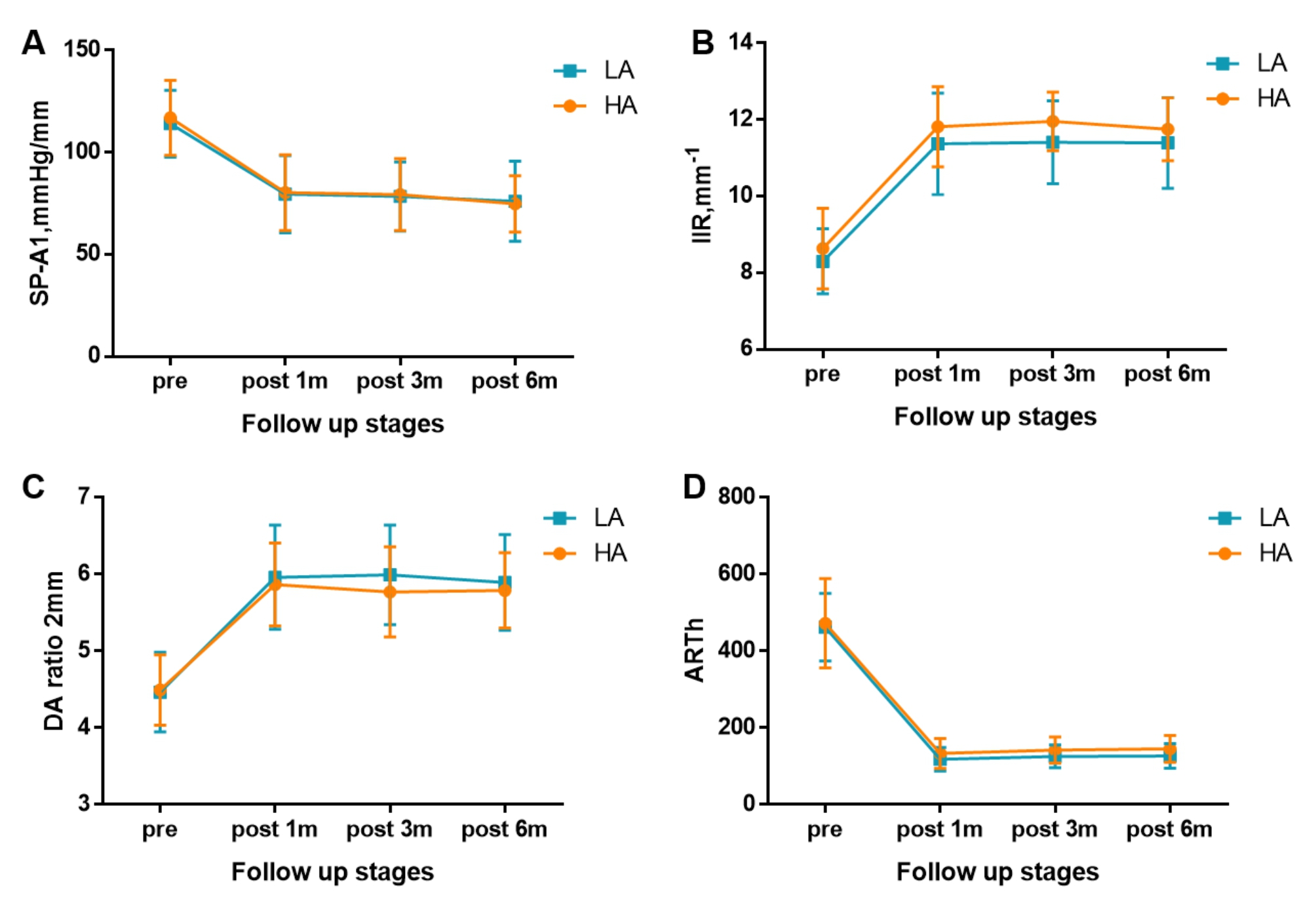
Changes in biomechanical parameters before and after FS-LASIK in both groups A: stiffness parameter at first applanation (SP-A1); B: integrated inverse radius (IIR); C: deformation amplitude ratio of 2 mm (DA ratio of 2 mm); D: Ambrosio relational thickness through the horizontal meridian (ARTh)

Table 4; Fig. 2 show the biomechanical parameters before and after surgery, Table 5 shows the changes (Δ) in biomechanical parameters, and Table 6 shows the multiple comparisons at each time point within each group.

**Table 4:** Results of repeated measurements analysis of variance in biomechanical parameters

Parameters	Group	Mean ± SD				*F* value			*P* value		
		Preoperative	1 month	3 months	6 months	Group	Time	Group*Time	Group	Time	Group*Time
SP-A1	HA group	116.966 ± 18.201	80.253 ± 18.328	79.304 ± 17.389	74.781 ± 13.665	0.073	265.705	0.540	0.788	< 0.001*	0.655
	LA group	114.073 ± 16.228	79.542 ± 18.733	78.377 ± 16.755	76.043 ± 19.438						
IIR	HA group	8.363 ± 1.039	11.815 ± 1.035	11.955 ± 0.757	11.749 ± 0.813	5.554	564.629	0.520	0.021*	< 0.001*	0.669
	LA group	8.297 ± 0.841	11.368 ± 1.312	11.409 ± 1.068	11.394 ± 1.168						
DA ratio 2 mm	HA group	4.494 ± 0.453	5.868 ± 0.535	5.771 ± 0.579	5.792 ± 0.483	1.045	314.398	1.684	0.309	< 0.001*	0.176
	LA group	4.466 ± 0.513	5.963 ± 0.671	5.995 ± 0.642	5.895 ± 0.618						
ARTh	HA group	472.645 ± 115.127	133.531 ± 38.539	142.370 ± 33.417	145.361 ± 34.920	3.540	1001.356	0.099	0.063	< 0.001*	0.960
	LA group	462.088 ± 87.111	118.264 ± 30.174	125.992 ± 29.340	126.918 ± 31.855						

SD, standard deviation; HA, high astigmatism; LA, low astigmatism; SP-A1, stiffness parameter at first applanation; IIR, integrated inverse radius; DA ratio 2 mm, deformation amplitude ratio 2 mm; ARTh, ambrosio relational thickness through the horizontal meridian. *P < 0.05

**Table 5:** Results of repeated measurements analysis of variance in changes of postoperative biomechanical parameters

Parameters	Group	Mean ± SD			*F* value			*P* value		
		1 month	3 months	6 months	Group	Time	Group*Time	Group	Time	Group*Time
Δ SP-A1	HA group	36.713 ± 14.709	37.662 ± 15.449	42.185 ± 16.236	0.917	4.509	0.298	0.341	0.012*	0.742
	LA group	34.531 ± 17.984	35.696 ± 16.193	38.030 ± 17.218						
Δ IIR	HA group	-3.180 ± 1.122	-3.319 ± 1.066	-3.113 ± 1.045	0.385	0.971	0.633	0.537	0.381	0.532
	LA group	-3.071 ± 1.084	-3.112 ± 0.715	-3.096 ± 0.761						
Δ DA ratio 2 mm	HA group	-1.374 ± 0.544	-1.278 ± 0.540	-1.298 ± 0.584	0.000	1.502	0.000	> 0.999	0.225	> 0.999
	LA group	-1.498 ± 0.531	-1.529 ± 0.536	-1.429 ± 0.459						
Δ ARTh	HA group	339.114 ± 111.576	330.275 ± 116.055	327.284 ± 117.542	0.088	13.690	0.301	0.767	< 0.001*	0.741
	LA group	343.824 ± 84.111	336.096 ± 79.417	335.170 ± 80.727						

SD, standard deviation; HA, high astigmatism; LA, low astigmatism; Δ SP-A1, changes of stiffness parameter at first applanation; Δ IIR, changes of integrated inverse radius; Δ DA ratio 2 mm, changes of deformation amplitude ratio 2 mm; Δ ARTh, changes of ambrosio relational thickness through the horizontal meridian. *P < 0.05

SP-A1 decreased at pos1m compared with the pre-surgery stage in both groups (both *P* < 0.001), indicating overall stiffness reduction, and then experienced non-significant fluctuations during follow-up (*P* > 0.05). Comparing pos1m and pos3m, Δ SP-A1 was larger at pos6m in both groups (*P* = 0.012). Neither SP-A1 (*P* = 0.788) nor Δ SP-A1 (*P* = 0.341) showed differences between the HA and LA groups.

**Table 6:** P value of Fisher’s LSD test of biomechanical parameters within HA and LA eyes

Group	Time	Time	SP-A1	IIR	DA ratio 2 mm	ARTh
HA	1	2	< 0.001	< 0.001	< 0.001	< 0.001
		3	< 0.001	< 0.001	< 0.001	< 0.001
		4	< 0.001	< 0.001	< 0.001	< 0.001
	2	3	0.982	0.701	0.701	0.075
		4	0.055	0.921	0.560	0.016
	3	4	0.232	0.317	0.997	0.893
LA	1	2	< 0.001	< 0.001	< 0.001	< 0.001
		3	< 0.001	< 0.001	< 0.001	< 0.001
		4	< 0.001	< 0.001	< 0.001	< 0.001
	2	3	0.957	0.990	0.979	< 0.001
		4	0.244	0.997	0.780	< 0.001
	3	4	0.677	0.999	0.559	0.923

1: preoperative; 2:1 month postoperative; 3:3 months postoperative; 4:6 months postoperative

SP-A1,  stiffness parameter at first applanation; IIR, integrated inverse radius; DA ratio 2 mm, deformation amplitude ratio 2 mm; ARTh, Ambrosio relational thickness through the horizontal meridian

IIR exhibited a significant increase from pre-to pos1m (both *P* < 0.001), demonstrating overall stiffness reduction, and remained stable during follow up. The variation in IIR was significant between the two groups (*P* = 0.021), but ΔIIR showed no difference (*P* = 0.537).

Another evidence of overall stiffness reduction was seen in the significant increases observed in the DA ratio of 2 mm at pos1m compared with the pre-surgery stage in both groups (both *P* < 0.001). A DA ratio of 2 mm then remained stable in all follow-up stages from pos1m to pos6m. Both the DA ratio 2 mm (*P* = 0.309) and Δ DA ratio 2 mm (*P* > 0.999) showed no difference between the HA and LA groups.

ARTh also significantly decreased, denoting overall stiffness reduction, in both groups from pre-to pos1m (both *P* < 0.001). ARTh then experienced a gradual, slight increase throughout the rest of the follow-up period in both of the groups (all *P* < 0.05). No significant differences in ARTh (*P* = 0.063) and ΔARTh (*P* = 0.767) were observed between the HA and LA groups at any time point during the preoperative and postoperative follow-up.

## Discussion

This study showed that FS-LASIK had relatively comparable UDVA, CDVA, efficacy, and safety in correcting high and low myopic astigmatism. The postoperative cylinder was significantly greater in HA group (-0.54 ± 0.39 D) than in LA group (-0.28 ± 0.26 D), which was similar to -0.46 ± 0.32 D 12 months after FS-LASIK in eyes with high astigmatism (≥ 2.0 D) [6]. The difference was also reported by Huang et al. [16] between the HA group (-0.68 ± 0.21 D) and LA group (-0.21 ± 0.17 D) 12 months after the SMILE procedure.

Many studies have found that corneal coma, spherical aberration, and total HOAs significantly increase after refractive surgery [10, 14, 17]. We all know that the corneal asphericity is significantly altered after corneal refractive surgery, and this morphological change causes the light through the peripheral cornea to focus earlier than the central cornea, thus showing an increase in postoperative corneal aberrations. These results were also observed in this study. We also found that the changes in spherical aberration and total HOAs were significantly lower in the LA group than in the HA group, whereas the changes in coma were similar between the two groups. The higher the degree of astigmatism, the deeper the peripheral cornea that needs ablation, and the steeper the transition from the ablation area to the non-ablation area of the cornea, which may cause a large change in the Q value, thus leading to an increase in HOAs of the cornea, especially spherical aberrations.

Several studies have shown a significant reduction of corneal biomechanical properties after different laser refractive surgeries [8, 9, 12, 13, 18]. However, the effect of different surgeries on corneal stiffness remains controversial. Guo et al. [18] reported that SMILE was superior to FS-LASIK and comparable to PRK He et al. [19] also demonstrated that SMILE had less effect on corneal biomechanics than FS-LASIK in high myopia; however, Raevdal et al. [20] did not find significant differences in a systematic review when comparing SMILE with flap-based procedures. Xin et al. [12] found that SMILE induced less corneal biomechanical degradation than FS-LASIK but more than tPRK in cases with comparable corrected refractive errors and optical zone diameter. A number of studies have reported that biomechanical changes induced by surgical operations are generally larger in the high myopia group than in the low-to-moderate myopia group [12, 13], which is expected as higher degrees of myopic correction typically require more tissue removal and hence can introduce larger reductions in corneal biomechanics. However, there are few reports on biomechanical changes in eyes with high myopic astigmatism after refractive surgery. In this study, the biomechanical impact of FS-LASIK in patients with high and low myopic astigmatism was evaluated by monitoring the changes in the in vivo biomechanical parameters obtained using the Corvis ST over a 6-month follow-up period.

The in vivo measurement of corneal biomechanics with air puff systems, such as the Corvis ST used in this study, has been assessed in earlier publications [21]. Among the several parameters that Corvis ST offers, four were selected for being directly associated with overall corneal stiffness, namely, SP-A1, IIR, ARTh, and DA Ratio 2 mm. A significant shift in parameter values towards softening was observed after surgery in both groups, and then experienced non-significant fluctuations during follow-up, except ARTh, which was continuously increased, but this change was small and insignificant when compared with the preoperative value.

SP-A1 and ARTh experienced a similar amount of reduction postoperatively in both groups, and the changes in IIR and DA ratio 2 mm values showed a similar increase in the HA and LA groups. Thus, we can infer that high astigmatism correction following FS-LASIK induced the same corneal biomechanical degradation as low astigmatism correction.

Refractive astigmatism is the sum of anterior corneal astigmatism (ACA) and intraocular astigmatism (ORA), which seems to be induced mainly by the posterior corneal surface and the lens, and vector analysis is a method to quantify ORA; however, it is well accepted that the main amount of astigmatism resulting from ACA and ORA is usually of low magnitude. Nevertheless, it has been reported that the efficacy of LASIK for low myopic astigmatism correction is lower in eyes with a high preoperative ORA [22]. In contrast, the ACA is the main source of astigmatism in eyes with high myopic astigmatism, and in these eyes, ORA did not seem to affect the visual and refractive results [23]. This study did not include any of these values or the figure of surgically induced change in astigmatism (SIA) / target-induced astigmatism (TIA), which was the first limitation of the study. In addition, there is no reference to pupil size, considering that spherical aberrations increase the diameter of the pupil preoperatively. In addition, we only evaluated changes in objective indicators, such as vision and aberrations, and did not investigate subjective visual quality indicators. Another limitation is the lack of retreatment rate data. Finally, further elaboration is necessary to evaluate the results, given the small sample size.

In conclusion, FS-LASIK had relative efficacy and safety in correcting high and low myopic astigmatism in this study, but with higher astigmatic undercorrection and a larger increase in spherical aberration and HOAs in HA eyes. The biomechanical degradation in patients with high astigmatism after FS-LASIK is similar to that in patients with low astigmatism, which also confirms the safety of FS-LASIK in correcting high astigmatism.

## Data Availability

All data generated or analyzed during this study are included in this published article.
